# Acute toxicity tests with *Daphnia magna*, *Americamysis bahia*, *Chironomus riparius* and *Gammarus pulex* and implications of new EU requirements for the aquatic effect assessment of insecticides

**DOI:** 10.1007/s11356-012-0930-0

**Published:** 2012-05-05

**Authors:** Theo C. M. Brock, René P. A. Van Wijngaarden

**Affiliations:** Alterra, Wageningen University and Research Centre, P.O. Box 47, 6700 AA Wageningen, The Netherlands

**Keywords:** Ecotoxicology, Aquatic invertebrates, Tier I, Micro-/mesocosms, Calibration

## Abstract

Threshold concentrations for treatment related effects of 31 insecticides, as derived from aquatic micro-/mesocosm tests, were used to calibrate the predictive value of the European Tier-1 acute effect assessment on basis of laboratory toxicity tests with *Daphnia magna*, *Chironomus* spp., *Americamysis bahia* and *Gammarus pulex*. The acute Tier-1 effect assessment on basis of *Daphnia* (EC_50_/100) overall was protective for organophosphates, carbamates and most pyrethroids but not for neonicotinoids and the majority of insect growth regulators (IGRs) in the database. By including the 28-day water-spiked *Chironomus riparius* test, the effect assessment improves but selecting the lowest value on basis of the 48-h *Daphnia* test (EC50/100) and the 28-day *Chironomus* test (NOEC/10) is not fully protective for 4 out of 23 insecticide cases. An assessment on basis of *G. pulex* (EC_50_/100) is sufficiently protective for 15 out of 19 insecticide cases. The Tier-1 procedure on basis of acute toxicity data (EC_50_/100) for the combination of *Daphnia* and *A. bahia* and/or *Chironomus* (new EU dossier requirements currently under discussion) overall is protective to pulsed insecticide exposures in micro-/mesocosms. For IGRs that affect moulting, the effect assessment on basis of the 48-h *Chironomus* test (EC_50_/100) may not always be protective enough to replace that of the water-spiked 28-day *C. riparius* test (NOEC/10) because of latency of effects.

## Introduction

**Fig. 1:**
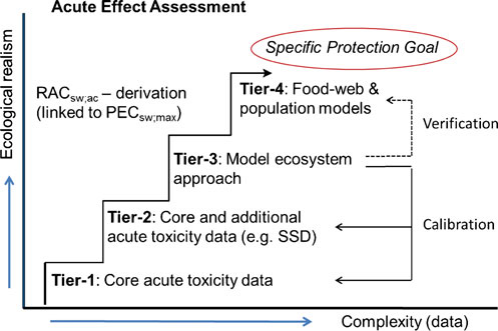
Schematic presentation of the tiered approach within the acute effect assessment for pesticides showing the refinement of the process through the acquisition of additional data and the possibility to use results of micro-/mesocosms (model ecosystem approach) to calibrate the lower tiers. *RAC*_*sw; ac*_ the Regulatory Acceptable Concentration for surface water within the context of the acute effect assessment scheme, *PEC*_*sw; max*_ the maximum concentration predicted for surface water, *SSD* Species Sensitivity Distribution approach (redrafted after Solomon et al 2008 and EFSA 2010)

Tiered approaches are the basis of environmental risk assessment schemes that support the registration of pesticides (e.g. Campbell et al. 1999; EC 2002; Boesten et al. 2007; Solomon et al. 2008). In this context, a Tier is defined as a complete exposure or affects assessment resulting in an appropriate predicted environmental concentration (PEC) or regulatory acceptable concentration (RAC). The concept of tiered approaches is to start with a simple conservative assessment and to do additional more complex work if necessary, but all tiers within the same scheme need to address the same specific protection goal (EFSA 2010; Nienstedt et al. 2011). This approach implies a cost-effective procedure both for industry and regulatory agencies. In the aquatic effect assessment Tier-1 normally is based on results of laboratory toxicity tests with a limited number of standard test species and the application of an appropriate assessment factor (AF). Subsequent higher tiers may include results of laboratory toxicity test with additional test species (allowing, e.g. the species sensitivity distribution (SSD) approach), aquatic micro-/mesocosm tests (model ecosystem approach), and ‘validated’ food-web and /or population models (Fig. 1). The tiered system as a whole needs to be: (1) appropriately protective, (2) internally consistent, (3) cost-effective and (4) address the problem with a higher degree of realism and complexity when going from lower to higher tiers. In pesticide risk assessment under Regulation (EC) no. 1107/2009 (EC 2009), the basic data requirement for the Tier-1 effect assessment are strictly defined (EC 2011). The current Tier-1 basic data requirement, however, are under discussion (see below), since the new Regulation 1107/2009 not only aims an appropriate protection of crops against harmful organisms but also a higher level of protection of the environment and non-target organisms than under the former Plant Protection Product Directive (91/414/EEC) (EC 1991).

A logical consequence of the principles of the tiered approach in the environmental risk assessment for pesticides is that results of an appropriate higher tier effect assessment may act as a reference to calibrate the lower tier effect assessment, because the assessment endpoint derived from a higher tier is closer to the actual objectives of the adopted protection goal (Fig. 1). In the aquatic effects assessment for pesticides, a micro-/mesocosm test may provide the appropriate higher tier effect endpoint when invertebrates or primary producers are at risk (Brock et al. 2006; Maltby et al. 2005, 2009; Van den Brink et al. 2006; Guy et al. 2011). In this paper, we therefore use threshold concentrations for treatment-related effects as observed in micro-/mesocosm studies treated with insecticides to calibrate the Tier-1 acute effect assessment procedure.

The main objective of this paper is to evaluate the Tier-1 effect assessment procedure for insecticides based on the current and the proposed new, but not yet adopted, EU dossier data requirement for acute toxicity to aquatic invertebrates. The risk assessment procedure on basis of the current data requirement is described in the EU Guidance Document on Aquatic Ecotoxicology (EC 2002) and in Commission Regulation No 544 (EC 2011). According to these documents, the acute insecticide risk to invertebrates is assessed by comparing the predicted peak concentration (PEC_max_) for edge-of-field surface water with the *Daphnia* 48-h EC_50_/100 value. In addition, according to EC (2002), an effect assessment on basis of the 28-day water-spiked *Chironomus* test in the presence of sediment (OECD 2004; Streloke and Köpp 1995) should be provided if the test substance concerns an insecticide with a specific toxic mode-of-action (e.g. neonicotinoid insecticide and insect growth regulator (IGR); *Daphnia* 48-h EC50 > 1 mg/L). In this 28-day water-spiked *Chironomus* test, the exposure concentration of the test compound is not maintained in the water compartment, and the effect endpoint (i.e. NOEC or EC_10_) is expressed in terms of the initial (nominal) exposure concentration in water. In the risk assessment on basis of this test the derived NOEC/EC_10_ is divided by an AF of 10, and the concentration thus obtained is compared with the PEC_max_. The Guidance Document on Aquatic Ecotoxicology (EC 2002) also states that for the acute effect assessment of insecticides like neonicotinoids and IGRs, a water-only acute *Chironomus* test may be appropriate, but that an official guideline for such a test needs to be developed.

For pesticide risk assessment under the new Plant Protection Product Regulation (EC 2009), commission regulation (EU) proposals circulate to revise the basic data requirement for the first-tier aquatic effect assessment. An important change in the proposed update is the inclusion of an acute test for a second aquatic arthropod species (besides *Daphnia*) as basic data requirement for insecticides and substances with insecticidal activity. The Mysid shrimp (*Americamysis bahia*) and/or larvae of the insect *Chironomus* spp. are mentioned as candidate. Since *A. bahia* is a saltwater crustacean, the insect *Chironomus* is considered a more relevant freshwater test species and an official OECD guideline for an acute aquatic tests with *Chironomus* has recently been published (OECD 2011; also see Weltje et al. 2010). Consequently, when in the near future the proposed new data requirement will be implemented, the acute toxicity to aquatic invertebrates will, in the first instance, be assessed on basis of (1) the acute laboratory 48-h EC_50_ for *Daphnia* (preferably *Daphnia magna*) and (2) the acute 48-h EC_50_ for *A. bahia* and/or *C. riparius* (or another *Chironomus* species mentioned in OECD (2011) such as *Chironomus dilutus* or *Chironomus yoshimitsui*). In the first-tier effect assessment an AF of 100 will be applied to the lowest acute EC_50_ value. The highest predicted peak concentration (PEC_max_) in edge-of-field surface water should not exceed the Tier-1 regulatory acceptable concentration (Tier-1 RAC) thus obtained. This risk assessment procedure is believed to sufficiently protect non-target invertebrates that dwell in edge-of-field surface waters from short-term exposures to insecticides.

In the current paper, we intend to evaluate the implication of the proposed new aquatic data requirement (inclusion of acute tests with *A. bahia* and/or *Chironomus* spp.) for the Tier-1 acute effect assessment for insecticides. In addition, we also explore whether the macro-crustacean *Gammarus pulex* is potentially a suitable standard test species for the risk assessment of insecticides since this species has been widely used in toxicity testing and is often used as focal species for developing ecotoxicological models (e.g. Galic et al. 2010; Ashauer et al. 2007, 2011).

## Materials and methods

Single-species acute toxicity data and micro-/mesocosm data were collected from existing open access toxicity data bases such as ECOTOX (www.epa.gov/ecotox/), Footprint (www.eu-footprint.org/ppdb.html), open “grey” literature including EU Draft Assessment Reports or DARs (http://dar.efsa.europa.eu/dar-web/provision ), RIVM reports (www.rivm.nl/bibliotheek/index-en.html), summary reports of EU member states (e.g. www.ctgb.nl) and scientific papers in the open literature (see Table 1). In addition, confidential data from industry was used that was provided to Alterra and used in the paper of Maltby et al. (2005). Insecticides were allocated to one of the following categories: organophosphates, carbamates, pyrethroids, insect growth regulators, neonicotinoids, biopesticides and other types of insecticides (Table 1).To respect the confidentiality of the data provided by industry, we made the different insecticides anonymous in the graphs but allocated them to one of the insecticide categories listed above. Criteria used to select single-species toxicity data were test endpoint and duration. Outliers where checked using original publications (i.e. sensu Maltby et al. 2005). Selected endpoints were the median effect concentrations for immobility or mortality observed in toxicity tests (EC_50_). The test duration selected was 48–96 h. Geometric means were calculated when more than one toxicity value was reported for the same endpoint of a species.

**Table 1:** Insecticides used for the evaluation and related scientific papers in the open literature that were consulted in addition to the open access toxicity data bases mentioned in the “Materials and methods” section

Group	Compound	Open literature references
Organophosphates	Azinphos-methyl	Van Wijngaarden et al. (2005a) and Maltby et al. (2005)
	Chlorpyrifos	Daam et al. (2008), López-Mancisidor et al. (2008), Maltby et al. (2005), Van den Brink et al. (1996), and Van Wijngaarden et al. (2005b)
	Fenitrothion	Van Wijngaarden et al. (2005a) and Maltby et al. (2005)
	Parathion-ethyl	Van Wijngaarden et al. (2005a) and Maltby et al. (2005)
	Phosalone	
	Phosmet	
Carbamates	Carbaryl	Ashauer et al. (2011), Maltby et al. (2005), and Van Wijngaarden et al. (2005a)
	Carbofuran	Van Wijngaarden et al. (2005a) and Maltby et al. (2005)
Pyrethroids	Cypermethrin	Maltby et al. (2005)
	Deltamethrin	Åkerblom et al. (2008), Beketov (2004), Maltby et al. (2005), and De Knecht and Van Herwijnen (2008)
	Esfenvalerate	Beketov (2004), Beketov and Liess (2008a), Lozano et al. (1992), Webber et al. (1992), and Van Vlaardingen et al. (2008)
	Fenvalerate	Van Wijngaarden et al. (2005a) and Maltby et al. (2005)
	Gamma-cyhalothrin	Van Wijngaarden et al. (2009) and Giddings et al. (2009)
	Lambda-cyhalothrin	Van Leeuwen et al. (2008), Maund et al. (2008), Roessink et al. (2005), Schroer et al. (2004), and Van Wijngaarden et al. (2006)
	Bifenthrin	
	Acrinathrin	
Benzylurea & other IGRs	Diflubenzuron	Brock et al. (2006) and Maltby et al. (2005)
	Novaluron	
	Teflubenzuron	Scheepmaker (2008a)
	Fenoxycarb	Smit and Vonk (2008)
	Pyriproxifen	Moermond (2008)
Biopesticides	Abamectin	Scheepmaker (2008b)
	Milbemectin	
Neonicotinoids	Clothianidin	
	Imidacloprid	Ashauer et al. (2011), Beketov and Liess (2008a), Posthuma-Doodeman (2008), and Stoughton et al. (2008)
	Thiacloprid	Beketov and Liess (2008b), Beketov et al. (2008), and Langer-Jaesrich et al. (2011)
	Thiamethoxam	
Other insecticides	Lindane	Brock et al. (2006) and Maltby et al. (2005)
	Methoxychlor	Brock et al. (2006) and Maltby et al. (2005)
	Flubendiamide	
	Spiromesifen	

Micro-/mesocosm data were used to derive safe threshold concentrations. Each study was classified into one of two exposure categories, namely (1) a single pulse exposure regime or (2) a repeated exposure regime. In addition, responses observed for the most sensitive endpoint of a study were ascribed to one of five effect classes (sensu Brock et al. 2006; De Jong et al. 2008). For each compound and exposure regime the NOEC_eco_ (=threshold concentration for treatment-related effects) was derived from test concentrations at which no statistical and ecological significant effects (Effect Class 1) or slight/transient effects on individual samplings only (Effect Class 2) were observed for the most sensitive population and/or community endpoint. When possible, for each compound a separate NOEC_eco_ data point for a single and a repeated treatment regime was derived. Construction of the NOEC_eco_ was as follows. In case only Effect Class 1 values were available, then this value was used as the NOEC_eco_. In case only Effect Class 2 values were available, then this value was divided by two ((Effect Class 2 concentration)/2) to estimate the NOEC_eco_. When both an Effect Class 1 and an Effect Class 2 value were available then the geometric mean of the Class 1 and Class 2 values was used as the NOEC_eco_. In case more Effect Class 1 values were available for a compound (e.g. from different micro-/mesocosm studies), then the highest of these values was used. In the same situation for Effect Class 2 values, the lowest Class 2 value was chosen.

Ecosystem threshold levels (NOEC_eco_) were then compared with arthropod first-tier Regulatory Acceptable Concentrations (=Tier-1 RACs) based on acute toxicity data for *D. magna*, *A. bahia, Chironomus* spp. and *G. pulex*. These Tier-1 RACs were obtained by dividing the acute toxicity values by an AF of 100 (i.e. (48–96 h E(L)C_50_)/100). In addition, the NOEC/EC_10_ values of the 28-day water-spiked *C. riparius* test was used to derive a Tier-1 RAC by dividing it by an AF of 10. Note that in the aquatic risk assessment for insecticides these Tier-1 RACs always are compared with the PEC_max_. NOEC_eco_ values for each insecticide were plotted against the RAC based on:

1. The acute toxicity (EC_50_) for *D. magna* and an AF of 100 (=Dm/100);
2. The NOEC/EC_10_ of the water spiked 28-day *C. riparius* test and an AF of 10 (=28dCr/10);
3. The lowest toxicity value from the acute EC_50_/100 for *D. magna* and the 28d-NOEC/10 for *C. riparius* (=Dm/100 and 28dCr/10);
4. The acute toxicity for *A. bahia* and an AF of 100 (Ab/100);
5. The acute toxicity for an OECD-*Chironomus* sp. (i.e. *C. riparius*, *C. dilutus* (=*Chironomus tentans*), *C. yoshimitsui* (OECD 2011)) and an AF of 100. When data were available for more than one species, the most sensitive was selected (=Chir/100);
6. The acute toxicity (EC_50_) for *G. pulex* and an AF of 100 (Gp/100);
7. The lowest acute toxicity value (EC_50_) from the *D. magna* and *A. bahia* tests and an AF of 100 (=(Dm & Ab)/100);
8. The lowest acute toxicity value (EC_50_) from the *D. magna* and OECD-*Chironomus* tests and an AF of 100 (=(Dm & Chir)/100);
9. The lowest acute toxicity value (EC_50_) from the *D. magna, A. bahia* or OECD *Chironomus* tests and an AF of 100 (=(Dm & Ab & Chir)/100).

These RACs were compared with the 1:1 Tier-1 RAC/NOEC_eco_ ratio. Compounds falling below the 1:1 line indicate that Tier-1 RAC values derived from single-species toxicity tests are protective of ecological effects towards arthropod communities in semi-field studies characterised by a single or repeated pulsed treatment regime.

## Results

**Fig. 2:**
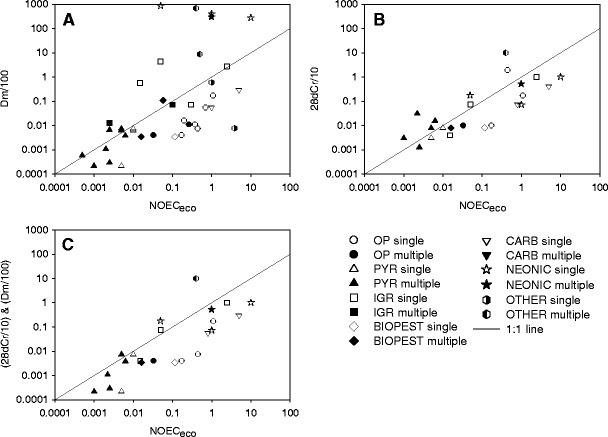
Plot of the acute Tier-1 RACs for insecticides against NOEC_eco_ values derived from aquatic micro-/mesocosm tests. The *line* represents the 1:1 ratio (RAC/NOEC_eco_). **A** RACs exclusively based on the acute toxicity data for *D. magna* (Dm/100); **b** RACs exclusively based on 28d NOEC/EC_10_ for *C. riparius* (28dCr/10); **c** RACs based on the lowest toxicity value of the combination acute EC_50_/100 for *D. magna* and the 28d- NOEC/10 for *C. riparius* (Dm/100 and 28dCr/10). *OP* organophosphates, *PYR* pyrethroids, *IGR* benzylurea/insect growth regulators, *NEONIC* neonicotinoids, *BIOPEST* biopesticides, *CARB* carbamates, *OTHER* other types of insecticides, *single* single insecticide application in micro-/mesocosm study, *multiple* repeated insecticide application in micro-/mesocosm study

Single-species acute toxicity data and NOEC_eco_ values could be compared for 31 insecticides which were categorized in one of seven groups (Table 1). The Tier-1 RAC based exclusively on the acute EC_50_ values for *D. magna* and the application of an AF of 100 was generally protective for organophosphates, carbamates and seven of the eleven pyrethroid cases (Fig. 2a). In contrast, similar RACs were not protective for any of the neonicotinoids evaluated since their *Daphnia* EC_50_/100 values were considerably higher (a factor of 28 to 17,020) than the NOEC_eco_, irrespective of exposure regime. Also three of the six IGR cases showed a RAC that was more than a factor of 5 to 10 greater that the line representing the 1:1 ratio. One biopesticide was a factor of 2 above this line as well as two compounds of the ‘other insecticides’ category by a factor of 17 to 1,718 (Fig. 2a).

RACs exclusively based on long-term toxicity data (28d NOEC) for *C. riparius* (water spiked test in the presence of sediment) and the application of an AF of 10 also appeared not to be sufficiently protective for eight of the twenty-three insecticide cases evaluated (Fig. 2b), although the deviations from the 1:1 line were less extreme (a factor of 2 to 25) than observed for the RAC based on acute toxicity of *Daphnia*. These eight insecticide cases comprised four pyrethroids and one organophosphate, IGR, neonicotinoid and ‘other insecticides’ each.

When Tier-1 RACs were derived on the basis of the lowest value from the 28d NOEC/10 for *C. riparius* and 48 h EC_50_/100 for *D. magna* (Fig. 2c) then the protection improved compared with the Tier-1 RACs based on the separate species (Fig. 2a, b). In Fig. 2c the Tier-1 RAC values for four of the 23 insecticide cases were a factor of 2 to 25 higher than their NOEC_eco_ values.

**Fig. 3:**
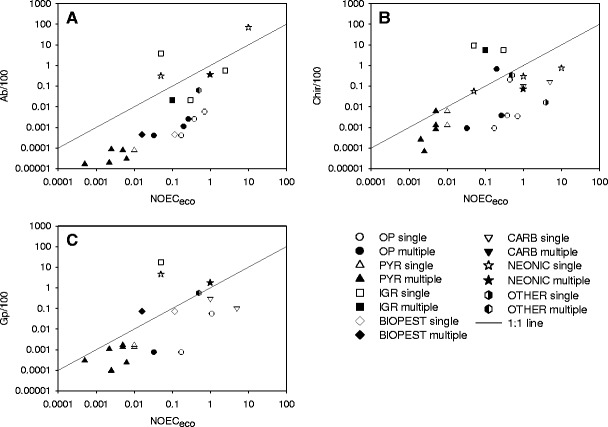
Plot of the acute Tier-1 RACs for insecticides against NOEC_eco_ values derived from aquatic micro-/mesocosm tests. The line represents the 1:1 ratio (RAC/NOEC_eco_). **a** RACs exclusively based on the acute toxicity for *A. bahia* (Ab/100); **b** RACs exclusively based on the acute toxicity for *Chironomus* (OECD species; Chir/100); **c** RACs exclusively based on acute toxicity data for *G. pulex* (Gp/100). For explanation of symbols, see Fig. 2

Tier-1 RACs exclusively based on the acute toxicity for *A. bahia* (EC_50_/100) resulted in three exceedences of the NOEC_eco_. The *A. bahia* EC_50_/100 value for an IGR was a factor of 75 higher than the corresponding NOEC_eco_ while that was a factor of 6 to 7 for two neonicotinoids (Fig. 3a).

Tier-1 RACs based on acute toxicity measured using the acute OECD *Chironomus* (EC_50_/100) test exceeded the NOEC_eco_ by a factor 19 to 185 for three IGR cases and a factor of 3 for an organophosphate (Fig. 3b). All nicotenoid cases are below the 1:1 line indicating that for neonicotenoids the acute *Chironomus* test is the best second Tier-1 test species, but less so for IGRs.

Tier-1 RACs based on the acute toxicity for *G. pulex* (EC_50_/100) resulted in exceedences of the NOEC_eco_ for two neonicotinoids (by a factor of 2 and 90) one biopesticide (by a factor of 5) and one IGR (by a factor of 350) (Fig. 3c).

**Fig. 4:**
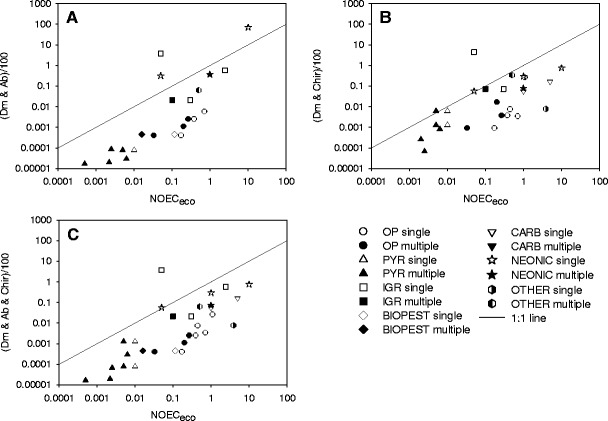
Plot of the acute Tier-1 RACs for insecticides against NOEC_eco_ values derived from aquatic micro-/mesocosm tests. The line represents the 1:1 ratio (RAC/NOEC_eco_). **a** RACs based on the lowest acute toxicity value for the combination *D. magna* and *A. bahia* ((Dm & Ab)/100); **b** RACs based on the lowest acute toxicity value for the combination *D. magna* and *Chironomus* (OECD species; (Dm & Chir)/100); **c** RACs exclusively based on the lowest acute toxicity data for the combination *D. magna*, *A. bahia* and/or *Chironomus* ((Dm & Ab & Chir)/100). For explanation of symbols, see Fig. 2

Tier-1 RACs (acute EC_50_/100) based on the most sensitive acute toxicity data for *D. magna* and *A. bahia*, showed a similar pattern of NOEC_eco_ exceedences (Fig. 4a) than when the effect assessment is based on acute toxicity of *A. bahia* alone (Fig. 3a), illustrating that in general the acute EC_50_ value for *A. bahia* is lower than that for *D. magna*.

Tier-1 RACs (acute EC_50_/100) based on the most sensitive acute toxicity data for *D. magna* and an OECD-*Chironomus* generally appeared to be protective for the insecticides investigated, except for the IGR fenoxycarb (Fig. 4b).

An overall high level of protection was achieved when deriving Tier-1 RACs (acute EC_50_/100) based on the most sensitive available acute toxicity value for *D. magna, A. bahia* and OECD-*Chironomus*, but again the Tier-1 RAC of one IGR (fenoxycarb) considerably exceeded its NOEC_eco_ (Fig. 4c).

## Discussion

Whether the Tier-1 effect assessment procedure on basis of the old data requirements (as presented in Fig. 2) is protective or not seems to depend on the specific toxic mode-of-action of the insecticide evaluated. In the case of organophosphates, carbamates and pyrethroids most Tier-1 RACs based on acute toxicity data for *D. magna,* or the combination of these data with the 28-d NOEC/EC_10_ data for *C. riparius*, are below the NOEC_eco_. However, the Tier-1 RACs for some other types of insecticides are above the NOEC_eco_, hence, not protective, particularly when these Tier-1 RACs are exclusively based on acute toxicity data for *D. magna*. Our evaluation clearly demonstrates that for insecticides with very specific modes-of-action, such as neonicotinoids and insect growth regulators, the concentration on basis of the EC_50_ of *Daphnia* and the application of an AF of 100 may not always protect sensitive invertebrates in micro-/mesocosm studies. Although by additionally using results of the 28-d water spiked *C. riparius* test (and application of the AF of 10) the Tier-1acute effect assessment considerably improves for most of the insecticides evaluated, this combination is still not fully protective for 4 out of 23 cases (for which both the 48-h EC_50_ data of *Daphnia* and the 28-day NOEC/EC_10_ data of *Chironomus* were available) (Fig. 2c).

The tier-1 effect assessment procedure on basis of the new data requirements (acute EC_50_ values for *D. magna* and *A. bahia* and/or *Chironomus*) appears to be protective for the vast majority of insecticides evaluated in micro-/mesocosms (Fig. 4). For the combination *D. magna* and *A. bahia* in 3 out of 22 cases the tier-1 RAC was not protective (Fig. 4a) while that was 1 out of 26 cases for the combination *D. magna* and *Chironomus* (Fig. 3b) and 1 out of 30 cases for the combination *D. magna* and either *A. bahia* or *Chironomus* (Fig. 4c).

The one case for which the Tier-1 RAC based on the proposed new data requirements was clearly not protective for effects observed in micro-/mesocosms concerned the IGR insecticide fenoxycarb. For this compound the Tier-1 RAC exceeded its NOEC_eco_ by a factor of 76 to 164 (Fig. 4a–c). The position of fenoxycarb in the plots has to do with the exceptionally broad range of Effect Class 2 concentrations (0.096–3.2 μg/L) reported in the cosm study available (in Smit and Vonk 2008). Note that we used the lowest Effect Class 2 value (0.096 μg/L) in our assessment. Moreover, the effects of fenoxicarb treatment observed in the cosms only involved single-event reductions in abundance of the cladoceran *Bosmina longirostris*. Clear long-term effects were reported at the next higher concentration of 11 μg/L (Smit and Vonk 2008). In addition, an experimental stream study, focussing on two mayfly populations, demonstrated a NOEC of 5 μg/L (Licht et al. 2004). This might suggest that the lowest Effect Class 2 concentration that we used to derive the NOEC_eco_ (by dividing this Effect Class 2 concentration by 2) for fenoxicarb was overly conservative.

The crustacean *G. pulex* is increasingly used as test species to underpin the aquatic effect assessment of pesticides, the advantages being that it is easy to handle in the laboratory and large enough to measure body burdens, and consequently, it is frequently used to study effects of time-variable exposures by means of toxicokinetic-toxicodynamic models (Ashauer et al. 2007, 2011). As for *D. magna*, Tier-1 RACs based on the acute toxicity of *G. pulex* are generally protective for neurotoxins (organophosphates, carbamates and pyrethroids), however, for more recently developed chemistries (e.g. neonicotinoids and IGRs) *G. pulex* may not be a representative sensitive species (Fig. 3c), although it is better than *Daphnia* (Fig. 2a).

Our evaluation demonstrates that the Tier-1, acute aquatic effect assessment on basis of laboratory EC_50_ values for *Daphnia*, *A. bahia* and/or *Chironomus* (EU commission regulation proposal for new data requirements) overall is protective for ecological effects due to pulsed insecticide exposures in the (semi-)field, in contrast to a Tier-1 effect assessment on basis of *Daphnia* alone. A lesson learned is that the Tier-1 effect assessment procedure needs to be critically evaluated/calibrated each time new insecticides with a novel toxic mode-of-action are placed on the market, e.g. by requiring not only the mandatory Tier-1 data but also an appropriate micro-/mesocosm study if read-across information is not available.

For neonicotinoids, and to a lesser extend IGRs, the key issue seems to be that crustaceans, and *D. magna* in particular, may be substantially less sensitive than aquatic insects (see e.g. Beketov and Liess 2008b). It can be argued that, based on the toxic mode-of-action of the active ingredient evaluated, Tier-1 insecticide studies always should include acute toxicity data for the most sensitive taxonomic group, which may be insects (e.g. the 48-h water only test with *Chironomus*). A comparison of the RACs for IGRs based on the 48-h acute *Chironomus* test (Fig. 3b) and the 28-day water-spiked *C. riparius* test in the presence of sediment (Fig. 2b), however, reveals that a test duration of 48 h may be too short to fully express the acute effects of IGRs. Also in mesocosm experiments latency of effects following short-term exposure to IGRs has been demonstrated (e.g. Brock et al. 2009).

A direct consequence of the possible new data requirements is that the Tier-1 effect assessment procedure becomes more conservative for those types of insecticides (e.g. organophosphates, carbamates, pyrethroids) that are already sufficiently covered by the Tier-1 RAC on basis of *Daphnia*. For example in Fig. 4c, that compares the NOEC_eco_ values with the acute Tier-1 RACs on basis of the new data requirements, the organophosphates and pyrethroids are on average a factor of 158 and 59 below the 1:1 line. This again may trigger more expensive higher-tier tests for these compounds. A possible solution to overcome unnecessary higher-tier testing is to calculate the Tier-1 RAC for insecticides by applying an AF of 100 to the geometric mean EC_50_ value (see EFSA 2005) of the available aquatic arthropod taxa in the core data set, if (1) sufficient read-across information for related compounds is available to underpin this approach and, (2) for the insecticide under evaluation the acute EC_50_ values for *D. magna* en *Chironomus* differ less than an order of magnitude. Alternatively, the default approach may be to apply the Geomean approach separately for crustaceans and insects and to select the lowest value. In a future paper we will calibrate the different options for the Geomean approach on basis of acute EC_50_ values for standard and additional arthropod test species and insecticides by comparing the RACs thus obtained with results of micro-/mesocosm tests.
